# RETRACTION: Extensive Analysis of Risk Factors Associated With Surgical Site Infections Post‐Cardiothoracic Open Surgery

**DOI:** 10.1111/iwj.70498

**Published:** 2025-04-02

## Abstract

**RETRACTION:** LiH. , ZhengX. , and GaoJ. , “Extensive Analysis of Risk Factors Associated With Surgical Site Infections Post‐Cardiothoracic Open Surgery,” International Wound Journal 21, no. 3 (2024): e14842, 10.1111/iwj.14842.38484717 PMC10940006

The above article, published online on 14 March 2024, in Wiley Online Library (http://onlinelibrary.wiley.com/), has been retracted by agreement between the journal Editor in Chief, Professor Keith Harding; and John Wiley & Sons Ltd. Following an investigation by the publisher, all parties have concluded that this article was accepted solely on the basis of a compromised peer review process. The editors have therefore decided to retract the article. The authors did not indicate their agreement with the retraction.

**RETRACTION:** H. Li , X. Zheng , and J. Gao , “Extensive Analysis of Risk Factors Associated With Surgical Site Infections Post‐Cardiothoracic Open Surgery,” International Wound Journal 21, no. 3 (2024): e14842, 10.1111/iwj.14842.38484717 PMC10940006

The above article, published online on 14 March 2024, in Wiley Online Library (http://onlinelibrary.wiley.com/), has been retracted by agreement between the journal Editor in Chief, Professor Keith Harding; and John Wiley & Sons Ltd. Following an investigation by the publisher, all parties have concluded that this article was accepted solely on the basis of a compromised peer review process. The editors have therefore decided to retract the article. The authors did not indicate their agreement with the retraction.

